# Assessment of knee laxity using a robotic testing device: a comparison to the manual clinical knee examination

**DOI:** 10.1007/s00167-015-3935-7

**Published:** 2015-12-24

**Authors:** T. P. Branch, S. K. Stinton, R. Siebold, H. I. Freedberg, C. A. Jacobs, W. C. Hutton

**Affiliations:** 1 University Orthopaedic Clinic, Decatur, GA USA; 2 ArthroMetrix, LLC, Atlanta, GA USA; 3 ATOS Klinik, Heidelberg, Germany; 4 Suburban Orthopaedics, Bartlett, IL USA; 5 Lexington Clinic, Lexington, KY USA; 6 Department of Orthopaedics, Emory University School of Medicine, Atlanta, GA USA

**Keywords:** Clinical knee examination, Knee injury, Knee laxity, Robotic knee testing, Ligament injury, Medial collateral ligament, Posterolateral corner

## Abstract

**Purpose:**

The purpose of this study was to collect knee laxity data using a robotic testing device. The data collected were then compared to the results obtained from manual clinical examination.

**Methods:**

Two human cadavers were studied. A medial collateral ligament (MCL) tear was simulated in the left knee of cadaver 1, and a posterolateral corner (PLC) injury was simulated in the right knee of cadaver 2. Contralateral knees were left intact. Five blinded examiners carried out manual clinical examination on the knees. Laxity grades and a diagnosis were recorded. Using a robotic knee device which can measure knee laxity in three planes of motion: anterior–posterior, internal–external tibia rotation, and varus–valgus, quantitative data were obtained to document tibial motion relative to the femur.

**Results:**

One of the five examiners correctly diagnosed the MCL injury. Robotic testing showed a 1.7° larger valgus angle, 3° greater tibial internal rotation, and lower endpoint stiffness (11.1 vs. 24.6 Nm/°) in the MCL-injured knee during varus–valgus testing when compared to the intact knee and 4.9 mm greater medial tibial translation during rotational testing. Two of the five examiners correctly diagnosed the PLC injury, while the other examiners diagnosed an MCL tear. The PLC-injured knee demonstrated 4.1 mm more lateral tibial translation and 2.2 mm more posterior tibial translation during varus–valgus testing when compared to the intact knee.

**Conclusions:**

The robotic testing device was able to provide objective numerical data that reflected differences between the injured knees and the uninjured knees in both cadavers. The examiners that performed the manual clinical examination on the cadaver knees proved to be poor at diagnosing the injuries. Robotic testing could act as an adjunct to the manual clinical examination by supplying numbers that could improve diagnosis of knee injury.

**Level of evidence:**

Level II.

## Introduction

Traumatic injuries in the knee are commonplace and account for more than 1.3 million emergency room visits and 19.4 million doctor office visits in the USA every year [47]. The likelihood of a successful recovery to a pain-free knee is greater with an early and accurate diagnosis of the injury followed by the appropriate diagnosis-led treatment protocol. Hitherto, the manual clinical knee examination has been the method used by examiners to assess the laxity of the knee and from that examination (often plus a radiograph and an MRI) make a judgment as to the best course of treatment. The results obtained from manual clinical knee examination are essentially subjective and are dependent upon the examiner’s training and experience [4, 30, 31]. Specific diagnosis of knee injuries based on manual clinical examination alone has been shown, in many cases, to be inconsistent and/or lacking in accuracy [2, 18, 20, 21, 23, 24, 26, 32, 33, 37–39, 43, 48].

In studies reporting on the use of the manual clinical knee examination, injuries to the posterior cruciate ligament (PCL), anterior cruciate ligament (ACL), or the meniscus are generally diagnosed more consistently and accurately as compared to those involving rotational or combined injuries. The ranges in diagnostic accuracy using the manual clinical knee examination have been reported as: medial meniscus tear 75–85 %; lateral meniscus tear 58–92 %; ACL tear 83–100 %; and PCL 96–100 % [20, 21, 26, 33, 39, 43]. However, other injuries such as medial collateral ligament (MCL) tears, posterolateral corner (PLC) injuries, or combined injuries have much lower ranges of accuracy [18, 23, 24, 32, 48]. For example, the posterolateral corner of the knee, which is a system of bones, muscles, tendons, and ligaments, has an anatomy that can vary from one person to the next. This variation can present difficulties when trying to identify rotational instability and injury patterns [2, 8, 23, 24, 37, 38].

A number of semi-automated devices such as the KT-1000 (MEDmetric, San Diego, CA), Genucom knee analysis system (FARO, Lake Mary, FL), Rolimeter (Aircast, Boca Raton, FL), Stryker knee laxity tester (Stryker Corporation, Kalamazoo, MI), and surgical navigation systems have been designed in an effort to obtain objective quantitative data in order to measure knee laxity (or knee stiffness) and to use the data (i.e. numbers) in the attempt to identify knee injuries [11, 17, 40, 46]. These devices have all provided some useful information. However, these devices are all semi-automated and they depend upon the examiner to control the direction and rate of the applied force. This could explain the fact that, when using semi-automated devices for diagnosis of knee injuries in a clinical setting, the relevant data obtained have not correlated well with patient satisfaction scores, functional scores, kinematic patterns, or the development of osteoarthritis after ACL reconstruction [9, 12, 22, 34, 42, 45].

Robotic knee testers, on the other hand, differ from the above-mentioned semi-automated devices in that the robot, once programmed, can standardize the magnitude and direction of the force applied and so provide vectors (numbers and direction) to give a less subjective measure of knee laxity. The advantage of this is that the test can be repeated using the same exact settings for direction and the rate of the applied force. Previous robotic systems include: a mechanized pivot shift device [29], the GNRB knee laxity testing device (GeNouRoB SAS, Laval, France) [25], the Robotic Knee Testing System (ERMI Inc., Atlanta, GA) ([3–7], United States Patent #’s: 7753862, 8753294, 8840570), and a device developed by Park et al. [35].

The purpose of this study was to collect objective quantitative data on knee laxity using a robotic testing device that is unique in its ability to test the knee in three planes: (1) anterior–posterior translation; (2) tibial axial rotation; and (3) varus–valgus rotation (RKT, ERMI Inc., Atlanta, GA). The data obtained from the device were compared to the results of manual clinical examination. The hypothesis of the study was that robotic testing of the knee could provide quantitative data on knee laxity that would indicate the injuries in cadaveric specimens, while manual clinical knee examination would be poor at diagnosing the injuries.

## Materials and methods

Two human cadaveric specimens were obtained for this study (see Table 1). Manual clinical knee examination and bilateral arthroscopic knee examinations were conducted on a number of cadavers in order to select specimens where the left and right knees on the cadaver were similar in terms of pre-existing conditions. The ligamentous structures were also judged to be intact prior to testing. If any ligamentous structure was damaged or if the pre-existing conditions were not equivalent between the left and right knee, then the specimen was excluded. After some care, two cadavers were selected that met the criteria. The pre-existing conditions noted during arthroscopic examination are shown in Table 2. Note that the pre-existing conditions are similar for the left knee and the right knee of each cadaver.

**Table 1:** Characteristics of the cadaveric specimens and details of the simulated injury for each specimen

Cadaver	Sex	Height (m)	Weight (kg)	Simulated injury
1	M	1.67	54.4	Left: grade III MCL injury
2	M	1.8	90.7	Right: grade III PCL corner injury

**Table 2:** Pre-existing conditions found in the cadaveric knees during arthroscopic evaluation, before simulated injuries had been inflicted on the left knee of cadaver 1 and the right knee of cadaver 2

Cadaver	Right	Left
1	Grade IV changes to MFC/PF, partial MM	Grade IV changes to MFC/PF, partial MM
2	Grade III changes to MFC, grade III changes to the PF, posterior horn of medial meniscus torn	Grade III changes to MFC, grade IV changes to the PF, posterior horn of medial meniscus torn

*MFC* medial femoral condyle, *PF* patellofemoral joint, *MM* medial meniscectomy

Under arthroscopic guidance, an injury was simulated in one knee of each cadaveric specimen. An isolated grade III MCL tear (complete ligament cut) was simulated in the left knee of cadaver 1, and a grade III PLC injury (capsule, popliteus tendon, and arcuate ligament were cut from the posterior lateral collateral ligament to the PCL) was simulated in the right knee of cadaver 2. The contralateral knee in each cadaver was left intact (i.e. with only the pre-existing conditions). However, a sham surgery was performed on each intact knee so that the injured knee could not be identified visually when the manual clinical knee examination was carried out. At the end of the experiment, complete sectioning of all four knees was carried out. This was done to confirm that the sham injuries (on the left knee of cadaver 1 and the right knee of cadaver 2) had been done correctly and that the uninjured knees (right on cadaver 1 and left on cadaver 2) aligned with the findings obtained arthroscopically as indicated in Table 2.

### Manual clinical examination

Without knowing that any of the knees had been injured in any way, five board-certified orthopaedic surgeons performed manual clinical knee examination on both knees of each cadaver. The pelvis of each cadaver was clamped to the examination table. Each participating examiner performed the following tests: Lachman-Trillat test, anterior drawer test, posterior drawer test, pivot shift test, varus and valgus stress tests at 0° and 30°, dial test at 30°, and anteromedial and anterolateral laxity tests (an anterior drawer test with the addition of a rotational force applied either medially or laterally). Each examiner was asked to report their results (or “opinions”) from each of the tests, and each examiner was asked to identify any particular injury on either knee. Laxity grades were assigned that represented the examiner’s opinion of the level of laxity for each test. The laxity grades reported in the tables are based on a system in which a grade 1 represents 0–5 mm or 0–5° of laxity, a grade 2 represents 6–10 mm or 6–10° of laxity, and a grade 3 represents >10 mm or >10° of laxity. Half numbers represent instances where the examiners were undecided between two grades (see Tables 3, 4, explained more fully in “Results” section below).

**Table 3:** Clinical grades for laxity and diagnoses of the left knee in cadaver 1 from the five board-certified examiners (BCE) for a series of manual clinical knee examinations

Examiner	Valgus test @0°	Valgus test @30°	Varus test @0°	Varus test @30°	Dial test @30°	Lachman	Pivot shift	Posterior drawer	Anterior drawer	Anterolateral	Anteromedial	Diagnosis
BCE1	0	1.5	0	1.5	0	0	0	0	0	1.5	0	Normal
BCE2	3	3	0	2	2	0	1	2	1	1	0	Torn PCL, LCL
BCE3	0	1.5	0	0	1	0	0	0	0	0	0	Partially torn MCL
BCE4	0	0	0	0	2.5	0	0	0	0	0	0	Posteromedial instability
BCE5	0	1	0	1	0	0	1	0	0	0	0	Normal

The left knee had received an isolated grade III MCL tear (complete ligament cut). The laxity grades are based on a system in which a grade 1 represents 0–5 mm or 0–5° of laxity, a grade 2 represents 6–10 mm or 6–10° of laxity, and a grade 3 represents >10 mm or >10° of laxity. Half numbers represented instances when examiners were undecided between two grades

**Table 4:** Clinical grades for laxity and diagnoses of the right knee in cadaver 2 from the five board-certified examiners (BCE) for a series of manual clinical knee examinations

Examiner	Valgus test @0°	Valgus test @30°	Varus test @0°	Varus test @30°	Dial test @30°	Lachman	Pivot shift	Posterior drawer	Anterior drawer	Anterolateral	Anteromedial	Diagnosis
BCE1	0	0	0	1.5	0	0	0	0	0	0	1.5	Torn PLC
BCE2	0	3	1	0	0	0	0	0	1	1	2	Torn MCL
BCE3	0	0	1.5	1.5	2.5	0	0	0	0	0	0	Torn PLC
BCE4	0	2.5	0	0	0	0	0	0	0	0	0	Torn MCL
BCE5	0	0	0	2	0	0	0	0	0	0	1	Torn MCL

The right knee had received a simulated grade III posterolateral corner injury (capsule, popliteus tendon, and arcuate ligament were cut from the posterior lateral collateral ligament to the PCL). The laxity grades are based on a system in which a grade 1 represents 0–5 mm or 0–5° of laxity, a grade 2 represents 6–10 mm or 6–10° of laxity, and a grade 3 represents >10 mm or >10° of laxity. Half numbers represented instances when examiners were undecided between two grades

### Robotic knee testing

**Fig. 1:**
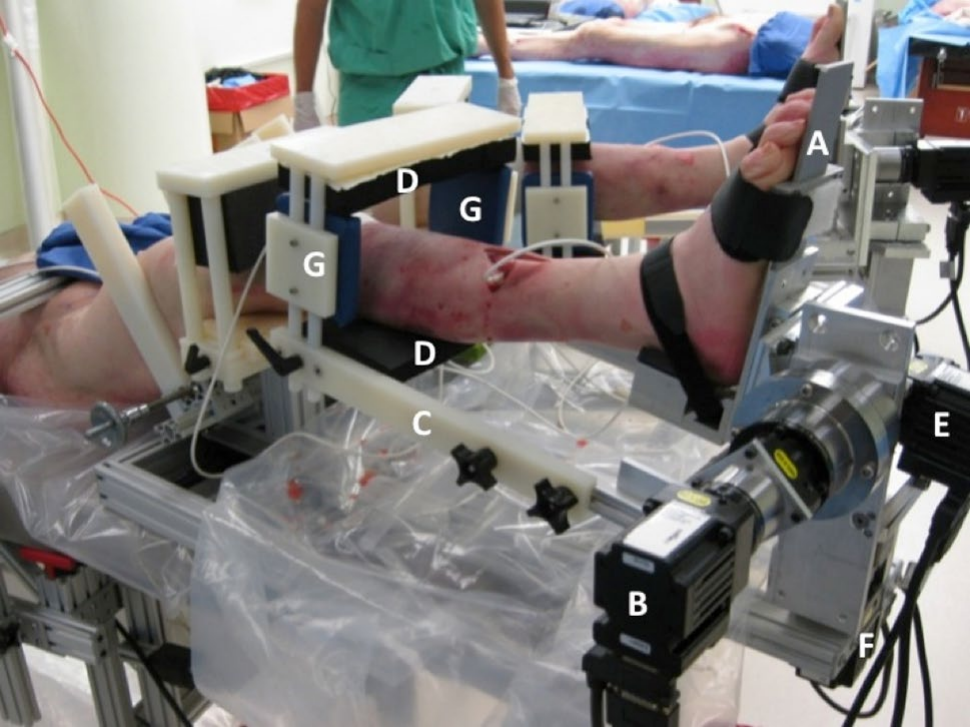
The robotic knee testing system (RKT) is shown with the feet of a cadaver strapped into footplates (*A*). Torque generated by the AP motor (*B*) during anterior–posterior testing was applied through the force application arm (*C*) by pads anterior to the shin and beneath the calf (*D*). Internal–external tibia rotation was achieved through torque generated by the rotation motor (*E*) which rotated the footplates. Torque generated by the VV motor (*F*) during varus–valgus testing was applied through the pads on the force application arm that are on either side of each leg (*G*)

For each cadaver, both knees were tested at the same time using the RKT device with the knees flexed to 30°. The cadavers were set up in the device by a third party blinded to the simulated injuries; this third party was not one of the examiners or the surgeon who had carried out the simulation of the injuries. Each femur was held fixed within the RKT device, and the tibia was manipulated by a footplate during internal–external tibial rotation testing and by an extendible force application arm during anterior–posterior (AP) and varus–valgus testing (see Fig. 1). Using the robot, the relative motion between the tibia and the femur was measured, or in other words, the laxity (or stiffness) of the knee. The robot also measured the force required to produce the motion between the tibia and the femur.

The RKT device performed three, single-axis laxity tests on each knee in the following planes: (1) anterior–posterior; (2) internal–external tibial rotation; and (3) varus–valgus. During AP testing, the RKT device first applied an anteriorly directed force at a constant displacement rate of 1 mm/s. The force was applied via pads below each calf that were attached to the force application arm. The applied force was adjusted based on each cadaver’s height and weight and represented 134 N in addition to the weight of the lower leg. This target of 134 N plus the weight of the leg was selected to match the force that is commonly applied during testing using the KT-1000 device during Lachman-Trillat testing (or anterior drawer testing) [11, 28]. Once the target applied force was achieved, the device reversed direction and pads on the force application arm pushed down on the shins until the same force was applied in the posterior direction. This test mimics the instrumented posterior drawer test using the KT-1000 device. The knees completed three AP preconditioning cycles followed by three test cycles. The three test cycles generally showed identical results, so the data were analysed from only one of them. The repeatability of the RKT device has been established in previous clinical studies where intra-class correlation coefficients ranged from 0.81 to 0.97 [4–6].

During internal–external tibial rotation testing, the footplates of the RKT device first moved into external rotation and then into internal rotation. This test mimics the dial test of the manual clinical knee examination. The torque threshold and angular velocity for rotational testing were chosen to be 6 Nm and 5°/s in this study. These values correspond to the values used by examiners during the dial test of the manual clinical knee examination in early pilot testing and match the 6 Nm used in other studies examining rotational laxity of the knee [1, 44]. As in AP testing, three preconditioning cycles were followed by three test cycles of internal–external rotation. Again, the three test cycles generally showed identical results, so the data were analysed from only one of them.

During varus–valgus testing, forces were applied through pads on the force application arm that were on either side of each leg. The legs were first manipulated into varus alignment until the torque threshold was reached in the varus–valgus motor. The motor then reversed directions until the same torque threshold was met while pushing the legs into valgus alignment. These tests mimic the varus and valgus test in the manual clinical knee examination. For varus–valgus testing, torque was applied at a constant velocity of 1°/s and the torque threshold was normalized to the cadaver’s height and weight and represented 1 Nm per unit body mass index (BMI) (Nm/kg-m^−2^). This amount of applied torque at the level of the pads on the force application arm was required to achieve 10 Nm of torque at the knee. These mimicked the values measured during varus and valgus stress tests of the manual clinical knee examination in pilot testing prior to this study and matched the force level at the knee used by Shultz et al. [41] in a study reporting on the reliability of measuring varus–valgus and internal–external laxity. As in AP testing, three preconditioning cycles were followed by three cycles of varus–valgus testing. Again, the three test cycles generally showed identical results, so the data were analysed from only one of them.

Torque values for all testing cycles were collected from the motors. Motion of the tibia relative to the femur was collected in 6 degrees of freedom (DOF) using an electromagnetic motion analysis system with sensors that were attached to the skin of the thigh and shin in each leg. Using 6 degrees of freedom sensors, the system is accurate to within 0.48 mm and 0.30° based on root mean square error (0.88 mm and 0.48° 95 % confidence interval) (Ascension Technologies, a subdivision of NDI, Bakersfield, CA, USA). The six DOF motion recorded during each test included three rotations (flexion–extension rotation, internal–external tibial rotation, and varus–valgus rotation) and three translations (anterior–posterior translation, medial–lateral translation, and distraction/compression). Torque–angular deformation curves were generated from the torque and motion data. Endpoint stiffness was calculated for each test using the slope of the last 20 % of the torque–angular deformation curve. A single examiner performed all robotic testing. No IRB approval was required for cadaveric testing at the Atlanta Medical Center where all testing was performed.

## Results

### Manual clinical examination results

The injured knees: the laxity grades and diagnoses reported by the examiners for the two injured knees (left on cadaver 1 and right on cadaver 2) are presented in Tables 3 and 4. Only one out of the five examiners correctly diagnosed the MCL injury in the left knee of cadaver 1. As shown in Table 3, the other examiners diagnosed the knee either as normal or as having a variety of other injuries including a tear of the PCL, LCL, or posteromedial instability. Two out of the five examiners correctly diagnosed the posterolateral corner injury in the right knee of cadaver 2 (see Table 4). The three remaining examiners diagnosed the injury as an MCL injury.

The “normal” uninjured knees: In this research report, we do not give detailed explanations on the examiner’s results for the uninjured knees (right knee in cadaver 1 and left knee in cadaver 2) as we wish to focus on the injured knees. However, as a brief summary, four of the five examiners diagnosed a ligament injury in the right uninjured knee in cadaver 1, including the two examiners that said the left injured knee was normal. In cadaver 2, four of the five examiners said that the left uninjured knee was normal, while the remaining examiner said that the left uninjured knee had a possible partial ACL rupture. These incorrect diagnoses in both the injured knees and the uninjured knees in each cadaver were possibly due to the laxity from pre-existing conditions in the knees.

### Robotic testing results

The results obtained from the robotic knee device during testing of cadaver 1 are shown in Table 5. During rotational testing, the medial translation of the tibia in the MCL-injured left knee of cadaver 1 was 4.9 mm greater than in the uninjured right knee (11.5 vs. 6.6 mm). During varus–valgus testing, the valgus angle of the MCL-injured left knee of cadaver 1 was 1.7° greater than in the uninjured right knee (9.2° vs. 7.5°). The MCL-injured knee also had lower endpoint stiffness when compared to the uninjured right knee during the valgus portion of varus–valgus testing (11.1 vs. 24.6 Nm/°), indicating that the injured MCL had a much softer endpoint than the MCL in the uninjured right knee. Results from robotic testing in other degrees of freedom reflected minimal differences between the knees (generally <1 mm or 1°).

**Table 5:** Laxity data measured from robotic knee testing comparing the MCL-injured left knee in cadaver 1 to the intact right knee

Cadaver 1	Rotation testing	Varus–valgus testing	
	Medial tibial translation (mm)	Valgus angle (°)	Valgus endpoint stiffness (N-m/°)
Right knee (intact)	6.6	7.5	24.6
Left knee (MCL injury)	11.5	9.2	11.1

Data from rotation testing and varus–valgus testing are reported. Endpoint stiffness is measured using the slope of the last 20 % of the load–deformation curve

The results obtained from the robotic knee device during testing of cadaver 2 are shown in Table 6. During rotation testing, the PLC-injured right knee of cadaver 2 had 3.9 mm less medial tibial translation than the uninjured left knee (2.9 vs. 6.8 mm). In the PLC-injured right knee of cadaver 2, the tibia showed 4.1 mm greater lateral translation (5.2 vs. 1.1 mm) when compared to the uninjured left knee during varus–valgus testing. The injured right knee also showed 2.2 mm greater posterior translation (5.4 vs. 3.2 mm) than the uninjured left knee during varus–valgus testing. Again, results from robotic testing in other degrees of freedom reflected minimal differences between the knees (generally <1 mm or 1°).

**Table 6:** Laxity data measured from robotic knee testing comparing the PLC-injured right knee in cadaver 2 to the intact left knee

Cadaver 2	Rotation testing	Varus–valgus testing	
	Medial tibial translation (mm)	Lateral tibial translation (mm)	Posterior tibial translation (mm)
Right knee (PLC injury)	2.9	5.2	5.4
Left knee (intact)	6.8	1.1	3.2

Data from rotation testing and varus–valgus testing are reported

## Discussion

The key finding in this study was that the results obtained using the robotic testing device showed that the device was able to provide objective numerical data that reflected differences between the knees in both cadavers suggesting which knee was injured and the type of injury. As expected and as noted in the previous literature, the examiners that performed the manual clinical examination on the cadaver knees proved to be poor at diagnosing the simulated injuries.

The poor results from the examiners that carried out the manual clinical examination were not unexpected in light of the lack of patient feedback and the inconsistency associated with the manual clinical knee examination that has been described in the literature. Yoon et al. [48] reported that the correct diagnosis of knee injuries was made in only 52 % of knees, incomplete diagnosis in 35 % of knees, and incorrect diagnosis in 13 % of knees. Oberlander et al. [32] reported similar findings with correct diagnosis being made in 56 % of cases, incomplete diagnosis in 31 %, and incorrect diagnosis in 13 %. The likelihood of an incorrect or incomplete diagnosis increased when two or more structures were injured, with correct diagnosis in only 30 % of these cases versus 70 % correct diagnosis when a single structure was injured [32]. The percentages of correct diagnoses in the current study are lower than those found in the Yoon and Oberlander studies; this could be due, in part, to the blinded nature of carrying out clinical examination on cadavers. In testing cadaveric knees, the examiner does not have patient input. The feel of laxity in cadaver knees could also be different than that seen in living patients.

The MCL injury in the left knee of cadaver 1 was correctly identified using the manual clinical knee examination by only one of the five examiners. The results from the robotic knee tests carried out on cadaver 1 showed that the difference in valgus laxity between the injured left knee and the intact right knee after the simulated MCL injury was a little over 1° (9.2° vs. 7.9°). This small side-to-side difference in valgus laxity would be very difficult to detect during a manual examination and was possibly one reason why the examiners found it difficult to diagnose the MCL injury correctly. The side-to-side differences in internal tibial rotation, medial tibial translation, and endpoint stiffness seen in the robotic testing results indicate an MCL injury even without a large difference in the valgus angles between the two knees. The endpoint stiffness of the injured knee was 13.5 Nm/° lower than of the intact knee. This concept of a soft versus hard endpoint in MCL injuries has also been described in numerous studies [13, 19, 27]. Robotic knee testing can yield an objective quantitative measure of the softness or hardness of the endpoint of a ligament. This objective quantitative measure of endpoint stiffness may provide a more consistent gauge of ligament integrity than the “endpoint feel” which is described in the manual clinical knee examination. The greater medial tibial translation in the injured knee in cadaver 1 is a characteristic of MCL injury; this has been reported by Frank et al. [14] in a study that measured kinematics in sheep knees after transection of the ACL and MCL.

The poor results in diagnosing the PLC injury using manual examination could be due to the fact that a change in the valgus angle of the knee can be attributed to causes other than an injury to the medial structures. PLC injuries can mask as MCL injuries due to similar global findings during the manual clinical knee examination (i.e. the feeling of increased valgus laxity). When performing the clinical valgus stress test, the lateral tibial plateau pivots about the lateral femoral condyle [10]. If the PLC is injured, the tibia can translate laterally and posteriorly on the femur; this gives the false impression of increased valgus rotation and MCL injury. The greater lateral translation and posterior translation of the PLC-injured left knee of cadaver 1 compared to the right knee (4.1 mm lateral and 2.2 mm posterior) were brought out in the robotic testing results. These findings match those found in previous studies where the posterolateral structures were cut in cadaver knees in order to measure the impact each structure had on the kinematics of the knee [15, 16, 36]. Unlike in the left knee of cadaver 1 which showed increased medial tibial translation compared to the normal knee, the right knee with the PLC injury in cadaver 2 showed greater lateral tibial translation. This larger lateral translation in the right knee of cadaver 2 suggests a lateral injury rather than a medial injury and can help differentiate between two conditions that can both give the impression of increased valgus laxity. Greater posterior translation in the injured knee compared to the intact knee can help differentiate a PLC injury from an isolated lateral collateral ligament injury.

This study was not without limitations. We only studied two cadavers (four knees). We examined numerous cadaver knees (alas, mainly old) to find knees that matched our selection criteria before finally selecting the two in our study. As with any cadaver study, the patient history was unknown and questions as to the condition of the knee could not be asked; thus, the manual examination was done blind. The cadaver specimens did have some previous knee issues including degeneration and meniscal damage (see Table 2). Ideally, to make our experiment cleaner, we would have preferred cadaveric specimens with no previous knee issues and minimal wear prior to the elicited injury. However, the vagaries of post-mortem collection did not allow this. In any case, existing knee issues have to be dealt with when performing a manual knee examination on patients in a normal clinical setting. The manual examination was also performed in cadaveric specimens rather than in living patients. Patient feedback, in terms of pain, subtle physical movements, or other complaints, can increase the accuracy of the manual examination. This feedback is not available in a cadaver study. However, in some situations, this information can be misleading. This study was able to show that quantitative knee laxity data could be obtained using robotic testing and that the data suggested the injuries in these two specimens. In the future, in vivo studies of patients with diagnosed knee injuries will be needed to validate a methodology for correlating quantitative knee laxity data to specific injury types.

The manual clinical examination is generally carried out at the first point of contact between patient and clinician. The manual clinical examination will never be set aside, since it can provide useful clinical information, especially with regard to the patient-reported history of the knee. However, useful clinical information can also be obtained from robotic knee testing. Robotic knee testing provides objective quantitative data that can aid the clinician in making a correct diagnosis. The manual clinical examination may also play an important role in providing insight into the interpretation of robotic testing results. The robotic data that are most relevant as determined by patient history and manual clinical examination (i.e. valgus extent and valgus endpoint stiffness for a suspected MCL tear) could be identified prior to the processing of the data. Data such as rotational laxity and endpoint stiffness can be used to differentiate between injuries such as an MCL tear and a PLC tear. In addition, robotic testing can provide additional information that clinicians can use to either confirm findings from the manual clinical examination or choose the appropriate tests from the manual clinical knee examination to perform again. In other words, the robot could act as an adjunct to the manual clinical examination by supplying numbers.

## Conclusions

The robotic testing device was able to provide objective numerical data that reflected differences between the injured knees and the uninjured knees in both cadavers. The examiners that performed the manual clinical examination on the cadaver knees proved to be poor at diagnosing the injuries. The diagnosis of knee injuries may be improved by the use of robotic testing as a supplement to the manual clinical examination.
